# A trivalent Apx-fusion protein delivered by *E*. *coli* outer membrane vesicles induce protection against *Actinobacillus pleuropneumoniae* of serotype 1 and 7 challenge in a murine model

**DOI:** 10.1371/journal.pone.0191286

**Published:** 2018-01-26

**Authors:** Kui Xu, Qin Zhao, Xintian Wen, Rui Wu, Yiping Wen, Xiaobo Huang, Yong Huang, Qigui Yan, Xinfeng Han, Xiaoping Ma, Yung-Fu Chang, Sanjie Cao

**Affiliations:** 1 Research Center of Swine Disease, College of Veterinary Medicine, Sichuan Agricultural University, Chengdu, China; 2 Sichuan Science-observation Experiment Station of Veterinary Drugs and Veterinary Diagnostic Technology, Ministry of Agriculture, Chengdu, China; 3 Department of Population Medicine and Diagnostic Sciences, College of Veterinary Medicine, Cornell University, Ithaca, NY, United States of America; 4 National Teaching and Experiment Center of Animal, Sichuan Agricultural University, Chengdu, China; University of Maryland, College Park, UNITED STATES

## Abstract

*Actinobacillus pleuropneumoniae* (APP) causes serious economic losses in the swine industry, and is the etiologic agent of porcine pleuropneumonia. In this study we have engineered a trivalent Apx fusion protein enclosed in outer membrane vesicles (Apxr-OMV) and studied its immunoprotective efficacy against APP serotypes 1 and 7 challenge in mice. The results showed that the IgG levels in the Apxr-OMVs immune group were significantly higher than those of the negative control (*P <* 0.05). Up-regulation of both Th1 (IFN-γ, IL-2) and Th2 (IL-4) cytokines were detected in splenocytes of Apxr-OMVs immune group. The survival rates 87.5% and 62.5% were observed against APP strain 1516 of serotype 7 and APP strain 2701 of serotype 1 in the groups of Apxr-OMVs immune group, respectively. Histopathological lesions of the pulmonary structure alveoli were found to be minimal in APX-OMV group challenged with APP serotypes 1 and 7. These results strongly indicated that engineered OMVs could effectively induce specific humoral or cellular immune responses. Moreover, Apxr-OMVs used as novel vaccine provides cross-protective immunity against different serotype 1 and 7 of APP infection in a mouse model. In contrast, the OMV-empty and PBS as negative controls or inactivated strain of APP-2701 and APP-1516 as positive controls for the animal study cannot provide protection or cross-protection.

## Introduction

*Actinobacillus pleuropneumoniae* (APP) is the causative agent of porcine pleuropneumonia (PCP), which is a member of the *Pasteurellaceaee* family and *Actinobacillus* category [1]. The disease is endemic throughout the world, inducing significant economic losses worldwide. APP contains two subtypes and 15 serotypes with apparent differences in virulence, based on variations in Capsular polysaccharide (CPS) and Lipopolysaccharide (LPS) [2].

The large number of serotypes and difficulties in discovering a vaccine providing significant cross-protection has hindered the development of an effective vaccine against APP [3]. Apx toxins belong to repeating structure toxin (RTX), and are one of the primary virulence factors of APP. It contains ApxI, ApxII, ApxIII, and/or ApxIV proteins [4–6],with different toxin combinations. Apx toxin synthesis and secretion relies on *ApxABCD* genes which encode ApxA, ApxB, ApxC, ApxD proteins, respectively [7, 8]. ApxIA, ApxIIA, and ApxIIIA, as APP toxin structure proteins, provide immune protection against APP infection with aluminum adjuvant [9,10]. ApxA contains many glycine-aspartate-rich nonapeptide repeat areas in the C-terminal part of the protein [5, 11, 12], which is the receptor binding domain [13].

Outer membrane vesicles (OMV) are spherical particles 20 nm to 250 nm in size and are constitutively released from the surface of Gram-negative bacteria [14]. The production of OMVs occurs when small portions of OM bulge away from the bacterial cells, pinch off, and release [15]. The potential functional role of OMVs are secretion of virulence factors, gene transfer (DNA or RNA), inter- and intracellular communication, biofilm formation, bacterial envelope stress relief, host immune modulation and host-pathogen interactions [16–18]. OMVs are composed primarily of phospholipids (PLs), OM proteins (OMPs), lipopolysaccharides (LPS) or lipooligosaccharides, periplasmic proteins and cell wall components [19]. OMVs are naturally enriched with immunogenic components, including outer membrane proteins (OMPs), periplasmic proteins, LPS, nucleotide acids, lipids, inner membrane proteins and cytoplasm proteins [20]. Many of these components contain pathogen-associated molecular patterns (PAMPs) detected by pattern recognition receptors (PRRs) such as Toll-like receptors (TLRs), and in conjunction with complement system activation drive inflammatory responses [21]. Therefore, OMVs are able to stimulate both innate and adaptive immunity *in vivo* and *in vitro* and have proven to be highly immunogenic and sufficiently potent to be effective vaccine components [22, 23]. It has been reported that the protein, fused with ClyA (a pore-forming hemolytic protein), expressed on the surface of OMVs and induce specific immune responses [23, 24]. In the present study, we engineer Apxr-OMV and its immunogenicity was assessed in a murine model.

## Materials and methods

### Bacterial strains, media and culture conditions

*E*. *coli* DH5α (Tiangen, China) was used as the host strain for cloning of plasmids. *E*.*coli* JC8031 (K12 Δ*tolRA*) was used as the host strain for vectors pBAD-18-CM expression and OMVs production [23]. *E*. *coli* strains were cultured in Luria-Bertani (LB) medium supplemented with 170 μg/ml chloramphenicol. Wild-type APP strain 2701 of serotype 1 and strain 1516 of serotype 7, isolated from pig farms located in Sichuan province, china in 2014 (reference), were used in the current study. APP strain 2 of serotype 2 and *E*.*coli* K-88ac31 were purchased from the China Institute of Veterinary Drug Control (Beijing, China).

APP was cultured in Tryptic Soy Agar or broth (TSA or TSB) (DIFCO Laboratories, USA) with 10% (v/v) calf serum (SIJIQING, China) and supplemented with 15 mg/ml nicotinamide adenine dinucleotide (NAD) (15 μg/ml) at 37°C.

### Plasmid construction for expressing the ClyA/Apxr fusion protein

**Fig 1:**
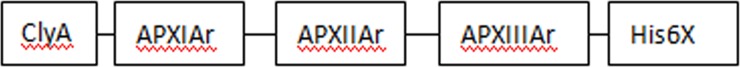
The structure of ClyA-Apxr. ClyA-Apxr constructs terminated by a 6x histidine-tag were expressed in *E*. *coli JC8031*, Apxr contains a fragment of ApxIAr, ApxIIAr and ApxIIIAr.

To express ClyA::ApxIAr::ApxIIAr::ApxIIIAr fusion protein (Apxr, Fig 1), a low-copy-number integrative vector, pBAD18-CM was engineered to express the fusion proteins. Primers used for amplifying the ClyA/pxIAr/pxIIAr/pxIIIAr genes were listed in Table 1, and the DNA sequences were shown in S1 Table. The resulting ApxIIAr/ ApxIIIAr PCR products were fused by seamless overlap extension PCR carrying an N-terminal histidine tag. Initially, DNA from *E*. *coli* K-88ac31 was used to amplify ClyA. The PCR product was then digested with SmaI and XbaI and cloned into pBAD 18-CM to make pBAD-ClyA. Primers targeting the ApxIAr gene were used on K-88ac31 DNA; this product was then digested (XbaI and SalI) and ligated into the pBAD-ClyA construct to make pBAD-ClyA-ApxIAr. A final PCR product ApxIIAr-IIIAr was generated (from strain 1516 DNA), digested with SalI/HindIII, and incorporated into the above construct to make the final fusion product, pBAD-APXr.

**Table 1:** Primers used to amplify and clone APP III gene sequences and *E*. *coli* ClyA sequences.

Gene	Primer sequences (5’-3’)
***ClyA*-F**	CGCCGAGCTCATGACTGAAATCGTTGCAGATA
***ClyA*-R**	TGCTCTAGAGACTTCAGGTACCTCAAAGAGTG
***ApxIAr*-F**	TGCTCTAGATATGCGGGTAACGGACATGATG
***ApxIAr*-R**	ACGCGTCGACAATATCCTTACCTAAACCACCGTAG
***ApxIIAr*-F**	ACGCGTCGACAATTTAGGTGCTGGTAACGATAATG
***ApxIIAr*-R**	TGCCAGTTTATCTTGTCCGC
***ApxIIIAr*-F**	CTCTGGCGGACAAGATAAACTGGCACATCTAGGCAATGGTAATGA
***ApxIIIAr*-R**	CCCCAAGCTTTTAGTGGTGGTGGTGGTGGTGAAAATCGCTACCATCGCCTCCT

The underline portions of the primer sequences were the restriction enzyme cutting sites (*ClyA*-F (forward): SacI; *ClyA*-R (reverse): XbaI; *ApxIAr*-F: XbaI; *ApxIAr*-R: SalI; *ApxIIAr*-F: SalI; *ApxIIIAr*-R: HindIII).

pBAD-APXr was electroporated into JC8031 and the transformants were selected on LB-chloramphenicol medium.

### Preparation of OMVs

OMVs were prepared as previously described [22]. Flasks containing 250 mL of medium were inoculated with 1 ml of overnight culture and allowed to grow at 37°C with shaking (200 rpm) until 0.5 at OD_600_, and then induced with 0.2% L-arabinose (Sigma, USA). Cultures were grown at 37°C for 12 h, centrifuged (5,000×g, 4°C, 10 min) to pellet bacteria and the culture supernatants were filtered through a 0.2 μm filter. Vesicles were isolated from the filtrates by ultracentrifugation (Beckman-coulter; Ti-32 rotor, 120, 000×g, 2.5h, 4°C), suspended in PBS, then stored at -20°C. The OMV-empties were prepared for control from JC8031-pBAD vector.

### Identification of engineered E. coli OMVs displaying Apxr

The expressed ClyA-Apxr fusion protein OMVs were analyzed by SDS-PAGE. Gray quantitative were analyzed by Image lab software (Bio-Rad). The relevant protein bands were purified directly from the gel. The samples were analyzed using Tandem mass spectrometry MALDI-TOF-TOF analysis, which was performed by Sangon Biotech (Shanghai Co., Ltd, China). The total protein concentration was determined by Pierce BCA protein Assay (Thermo Fisher Scientific, USA).

The Apxr-OMVs were visualized using electron microscopy as described [23], briefly vesicles were negatively stained with 2% phosphotungstic acid on 400-mesh Formvr carbon-coated copper grids and viewed in a FEI Tecnai G^2^ F20 transmission electron microscope.

To demonstrate the distribution of ClyA-Apxr, Apxr-OMVs were treated with 100 μg/ml proteinase K (PK, Tiangen) for 8 h at 37°C to degrade surface-exposed proteins. 0.1 mol/L Ethylene Diamine Tetraacetic (EDTA, Tiangen) was used to open the OMV structure to release the intracellular protein. The processed OMVs were used to immunologically detect Apxr protein via Western-Blot as described using anti-His6x primary antibody (Abmart, USA).

### Ethics statement

All experiments involving animals were conducted in compliance with the Animal Welfare Act and related regulations of Sichuan Agricultural University related to animal experiments (Cheng’du, china; Approval No.2011-028). All animal protocols were approved by the committee of Sichuan Agricultural University. The principles stated in the Guide for the Care and Use of Laboratory Animals were followed. All efforts were made to minimize animal suffering during the experiments. Mice were housed with food and water *ad libitum* and monitored under the care of full-time staff and in accordance with the rules of the Veterinary Medical College at Sichuan Agricultural University. All animals were acclimated for 1 week prior to experimentation. Humane endpoints were observed and utilized over the entire experimental period. Mice that were either unable or unwilling to eat and/or drink during the animal experiments and all the remaining Mice at the end of animal experiments were euthanized immediately by CO_2_ by a trained technician and approved by the EAWC.

### Immunization and challenge

Four to Five week females BALB/c mice (12–14 g) were purchased from Chengdu Dossy Experimental Animal Co., Ltd. Mice were divided randomly into five groups and were vaccinated subcutaneously. For the initial vaccination, Group 1 was vaccinated with Apxr-OMV (200 μl, 80 μg, n = 16) containing ~ 0.5 μg total Apxr antigen; group 2 with OMV-empty (200 μl, 80 μg, n = 16); groups 3 4 were vaccinated with inactivated APP strain 2701 (200 μl, 1×10^9^ CFU; n = 16) and strain 1516 (200μl, 1×10^9^ CFU; n = 16) with equal volume of aluminum adjuvant (alum, Sigma, USA), respectively; group 5 was injected with 200 μl PBS as a negative control. The immunization was performed twice on days 0 and 14, with identical dosages. None of the mice died throughout the immunization study. Before immunization and two weeks post booster, blood samples were obtained by tail vein bleeding. To get an accurate challenge dosage, the LD_50_ of APP strain 2701 and strain 1516 were determined using a Reed-Muench method (24). Two weeks after second immunization, three mice were randomly selected from each group for a Lymphocyte proliferation assay as previously described (25). Each group was randomly selected into two subgroups (n = 8); these were intraperitoneally challenged (10 LD_50_) with approximately 2.3 × 10^7^ CFU or 1 × 10^9^ CFU APP strain 2701 or 1516, respectively. The mice were carefully monitored daily after challenge for the presence and severity of respiratory symptoms and general illness or mortality for seven days. Mice were euthanized by CO_2_, when they were moribund. lung tissues were collected for histopathologic analysis. The mortality of mice was observed and recorded over a post-challenge period of seven days.

### Detection of antibody by enzyme-linked immunosorbent assay (ELISA) and cytokine expression levels in the immune serum

The antibody levels were detected by the indirect enzyme-linked immunosorbent assay (ELISA) as previously described with slight modifications [25]. Briefly, 96-well plates (Costar, USA) were coated with purified recombinant protein [200 ng/100 μl diluted in 0.02 M carbonate-bicarbonate buffer (PH 9.6)] and incubated overnight at 4°C. Wells were subsequently washed thrice with PBST, then incubated with 5% (w/v) bovine serum albumin (BSA) in PBST for 1.5 h at 37 h, followed by washing(3x). Plates were incubated with 100 μl sera diluted in 1:100 for 1.5 h at 37 h. PBST was used to wash the plates (4x); biotinylated goat anti-mouse HRP-IgG (1:5000, Bryotime, China) was then added to the wells (100 μl) and plates were incubated for 1 h at 37 h. After washing 5 times with PBST, TMB was added to the plate wells and kept for 10 min in a dark room. The reaction was stopped by adding 50 μl of 2 M H2SO4 to each well. Absorbance (OD) of the plate wells was read at a wavelength of 450 nm using a spectrophotometer (Bio-Rad).

The levels of IFN-γ (Interferon γ), IL-2 (Interleukin 2) and IL-4 (Interleukin 4) in the immune serum were determined using mouse ELISA kit (Elabscience, China), according to the instructions of manufacturer. The absorbance of the plate wells was read at a wavelength of 450 nm using a spectrophotometer (Bio-Rad).

### Lymphocyte proliferation assay

Lymphocyte proliferation assay was performed as previously described with minor modifications [26]. Splenocytes were prepared as previously described [27, 28]. Samples were centrifuged 5 min at 300 rpm/ min and splenocytes were suspended with RPMI incomplete medium. Splenocytes suspensions were processed using Red Blood Cell Lysis Buffer according to manufacturer’s instructions (SOLARBIO, china). Splenocytes were washed three times with Hank’s Balanced Salt Solution (HBSS) (THERMO, USA) and suspended in complete RPMI medium (THERMO, USA). Splenocyte density was adjusted to 5 × 10^5^ cell per ml, and 100 μl of this suspension was added to each well of a 96-well culture plates (COSTAR, USA). The cells were stimulated with Apxr-OMVs (5 μg/ well) or concanavalin A (ConA) (SIGMA-ALDRICH, USA), then incubated for 72 h at 37°C in a 5% CO_2_ incubator. Lymphocyte proliferation assay was performed using MTS Cell Proliferation Assay Kit (BEYOTIME, China), following the manufacturer’s instructions. MTS solution was added to the wells; plates were incubated for another 4 h. Absorbance following the incubation was measured at 490 nm using a microplate reader (Bio-Rad, USA).

### Histopathology

Lung samples were collected and fixed in 10% neutral formalin solution. Lung tissues were sectioned into 5 μm thick sections, and HE-stained for evaluation of histopathological changes viewed under an Olympus DP71 microscope [28].

### Statistical analysis

The data were statistically analyzed using the GraphPad Prism (GraphPad Software, USA) and SPSS 19.0 software using Student’s *t*-test for the comparison of the different treatment groups. *P*-values of < 0.05 were considered as significantly different and were represented with asterisk. *P* -values of < 0.001 were represented with two asterisks.

## Results

### Exogenous Apxr protein was successfully displayed on derived OMVs

**Fig 2:**
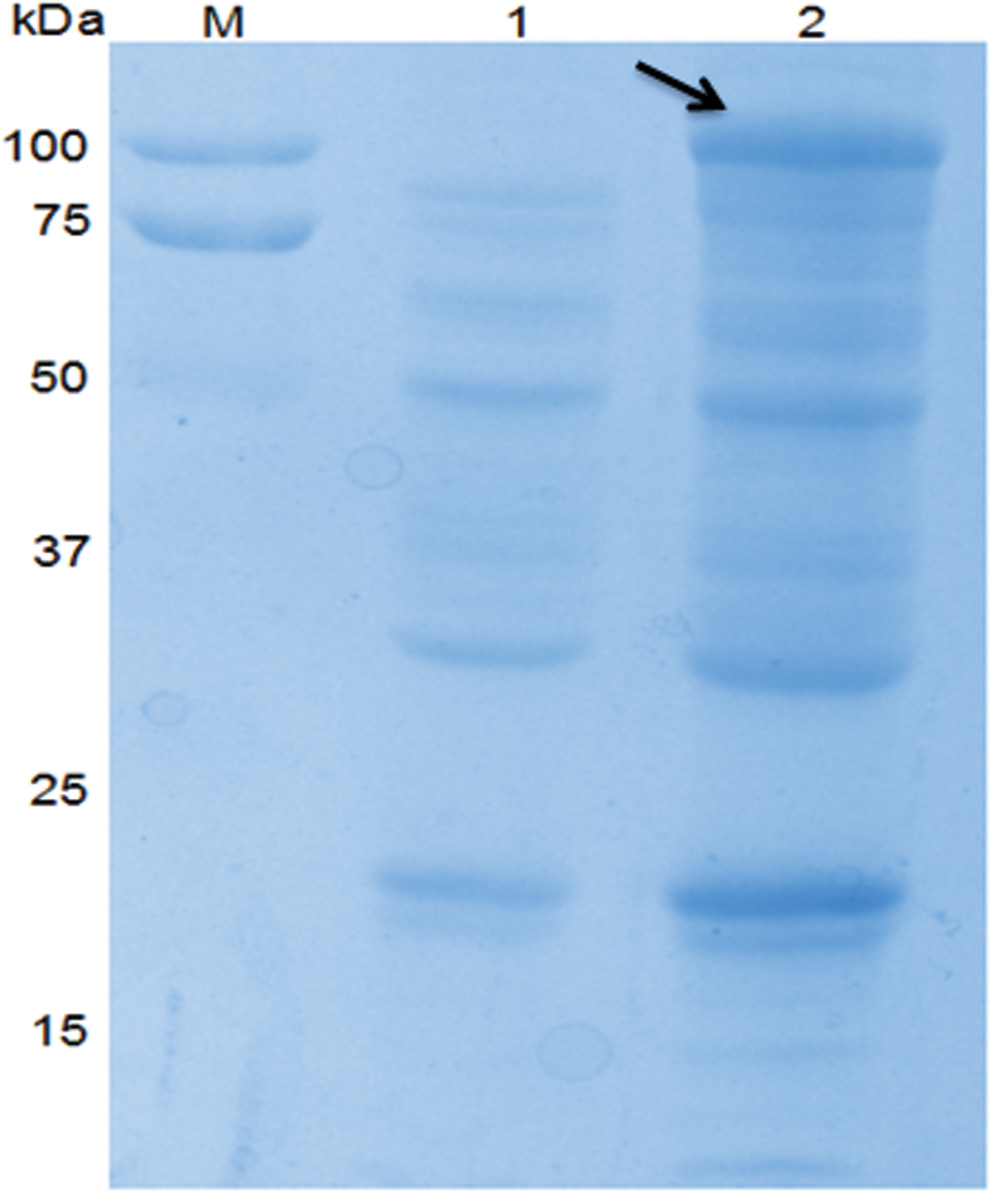
SDS-PAGE of JC8031 recombinant strain. Lane M: protein marker. Lane 1: JC8031-pBAD. Lane 2: JC8031- pBAD -Apxr.

**Fig 3:**
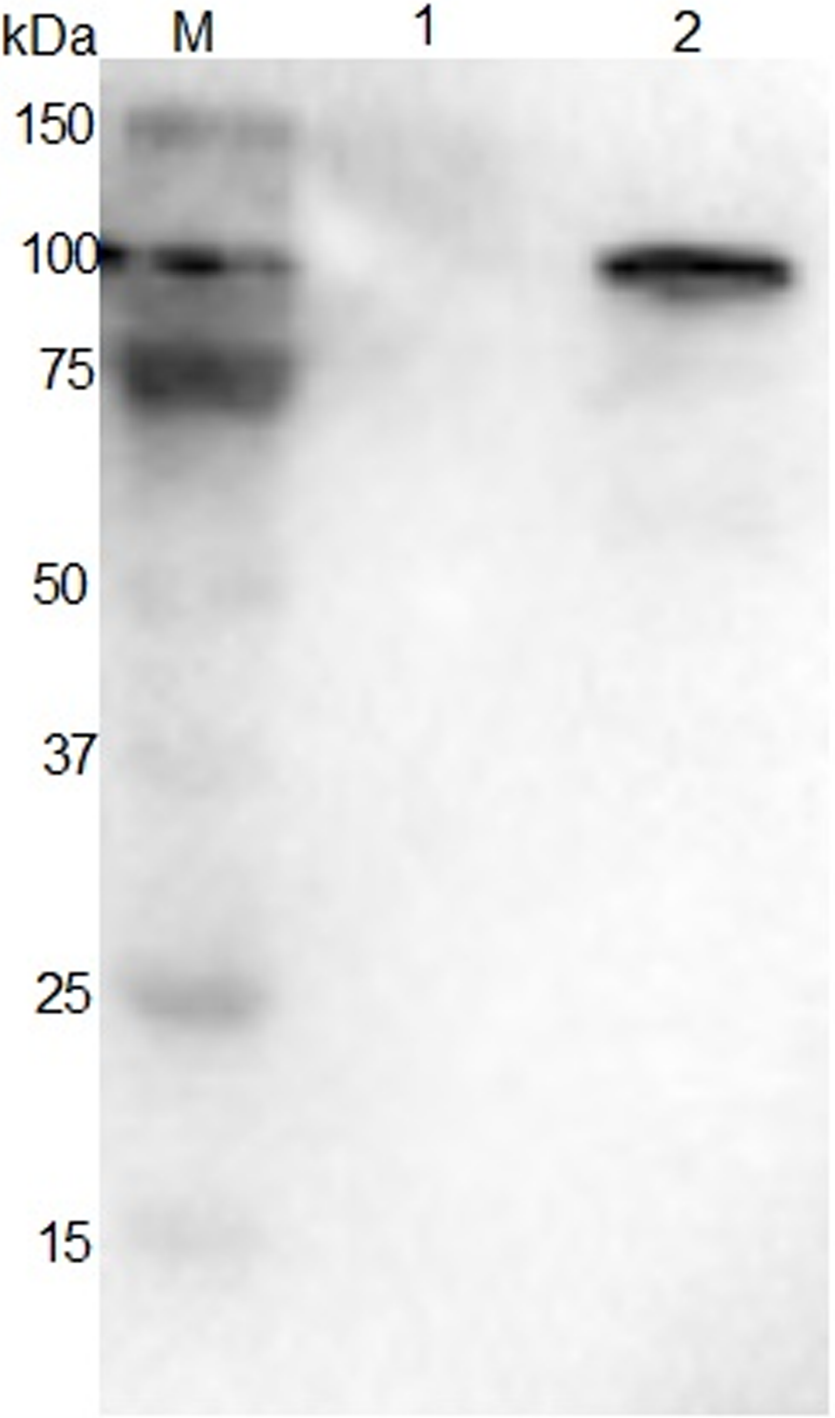
Western blot of JC8031 recombinant strain reacted with corresponding mouse anti-sera. Lane M: protein marker. Lane 1: JC8031- pBAD. Lane 2: JC8031- pBAD -Apxr.

**Fig 4:**
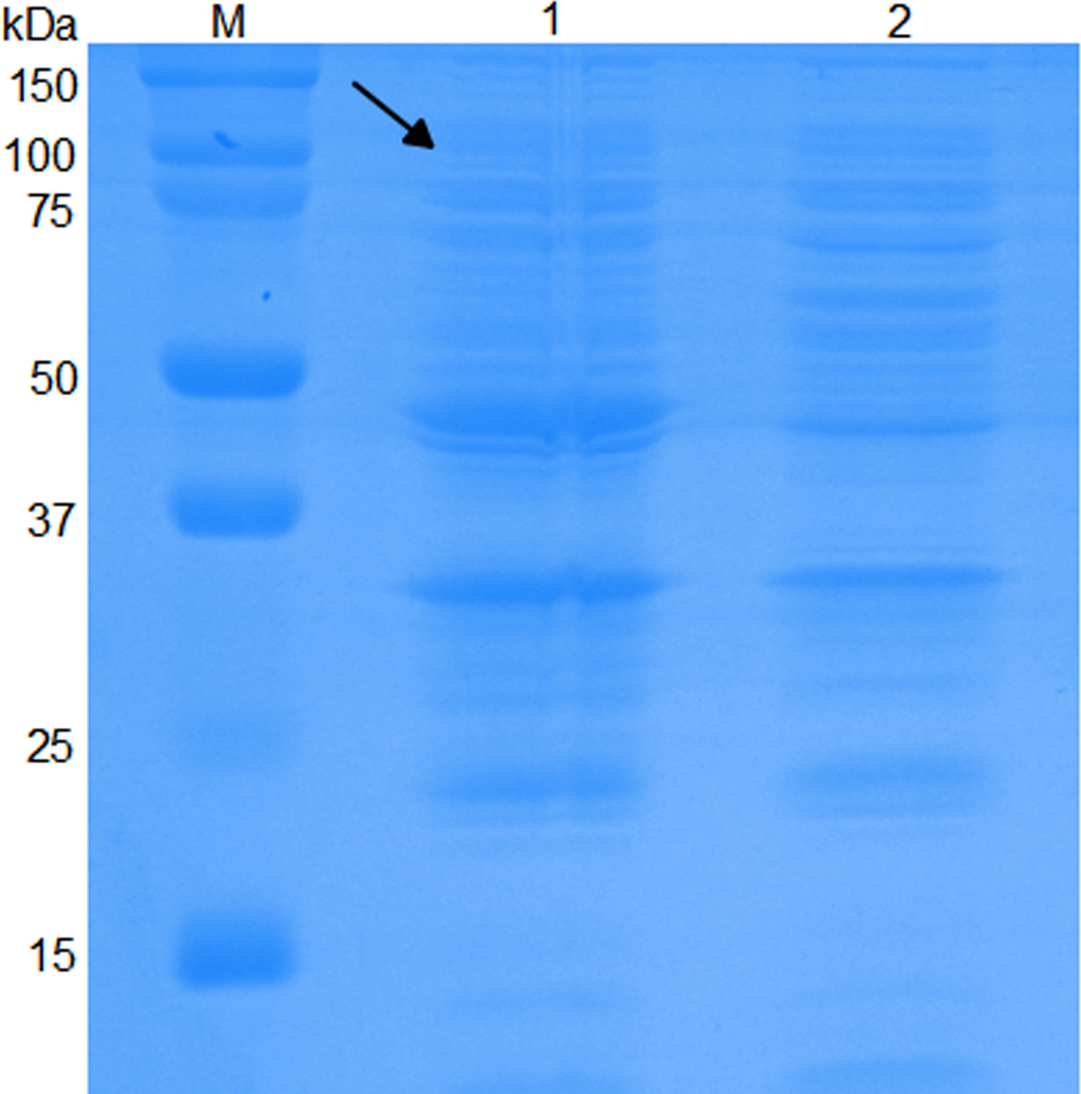
SDS-PAGE of Apxr-OMVs recombinant proteins. Lane M: protein marker. Lane 1: Apxr-OMV. Lane 2: OMV-Empty.

**Fig 5:**
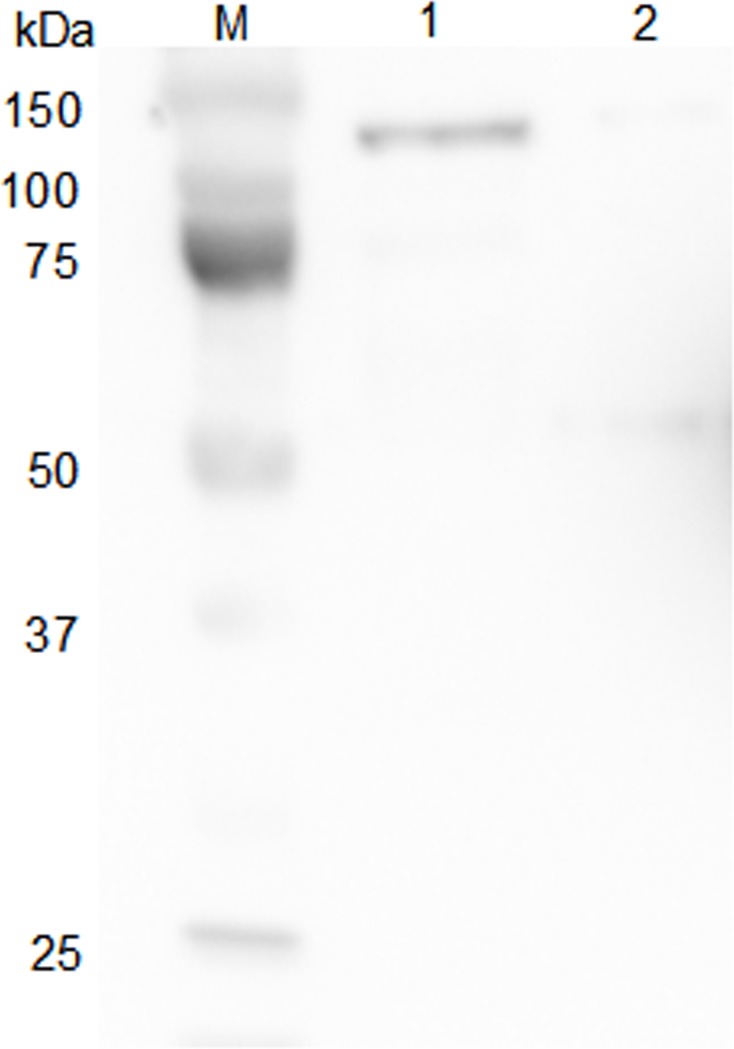
Western blot of OMVs proteins reacted with corresponding mouse anti-sera. Lane M: protein marker. Lane 1: Apxr-OMV. Lane 2: OMV-Empty. The arrow indicates the destination band.

As shown by SDS-PAGE and Western-Blot, the sample of preparative Apxr-OMVs after induction with 0.2% L-arabinose showed the appearance of an obvious band of approximately 100 kDa in comparison with preparative OMV-empty samples, indicating the successful expression of the ClyA-Apxr fusion protein (Figs 2–5).

**Fig 6:**
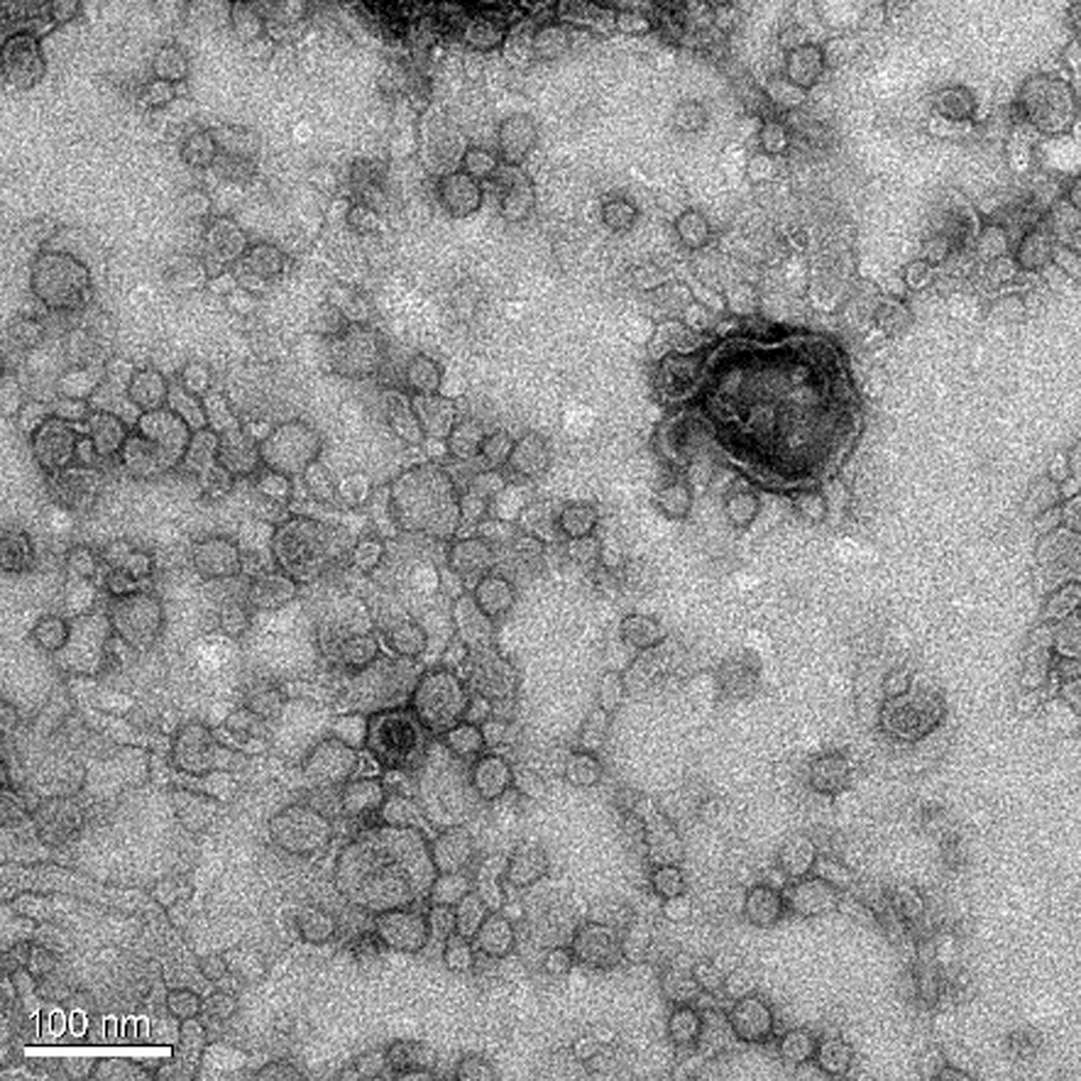
Transmission electron microscope image of wt-OMVs. The bar indicates 100 nm.

**Fig 7:**
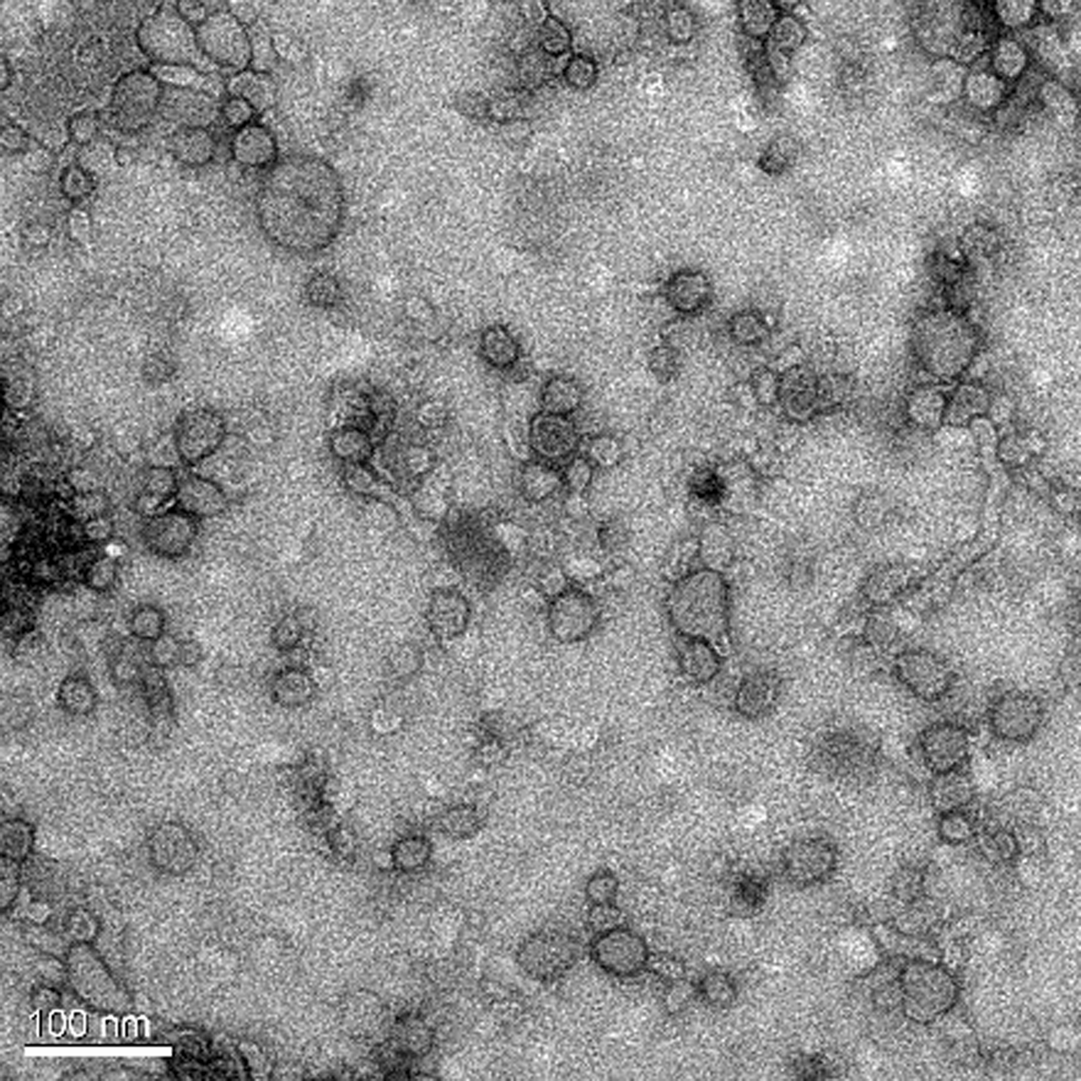
Transmission electron microscope image of recombinant Apxr-OMVs. The bar indicates 100 nm.

Electron microscopy image showed the spherical bilayer structure of OMVs (Figs 6 and 7), and the morphology of OMVs-empty and Apxr-OMVs was similar. This indicated that engineered Apxr-OMVs maintained the structure of natural vesicles. Software analysis showed that the largest OMVs diameter was 60 nm, a figure close to that measured for the OMV-empties (100 nm).

**Fig 8:**
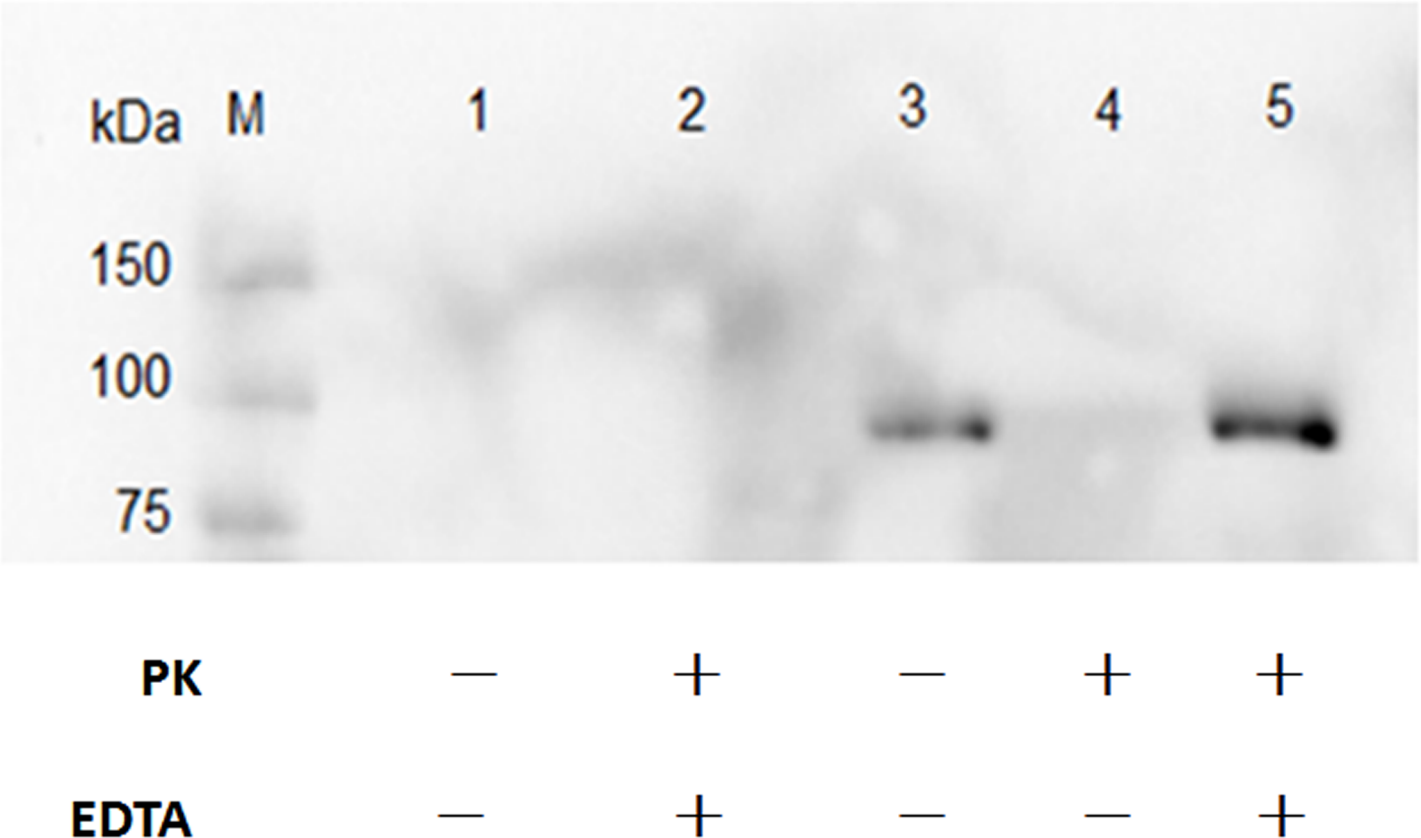
Demonstrate the distribution of ClyA-Apxr in Apxr-OMVs. Lane M: protein marker. Lane 1: the OMVs-empty. Lane 2: proteinase K plus EDTA treated Apxr-OMVs. Lane 3: untreated Apxr-OMVs. Lane 4: proteinase K treated Apxr-OMVs. Lane 5: EDTA treated Apxr-OMVs.

Immunoblotting analyses using anti-His6x primary antibody showed that there were no specific band on the proteinase K treated Apxr-OMVs and proteinase K or Ethylene Diamine Tetraacetic (EDTA) treated Apxr-OMVs, however the specific band present to untreated Apxr-OMVs and EDTA treated Apxr-OMVs (Fig 8). This result indicated that ClyA-Apxr was likely to be displayed on the surface of the engineered Apxr-OMVs.

### Detection of antibody and cytokine levels

**Fig 9:**
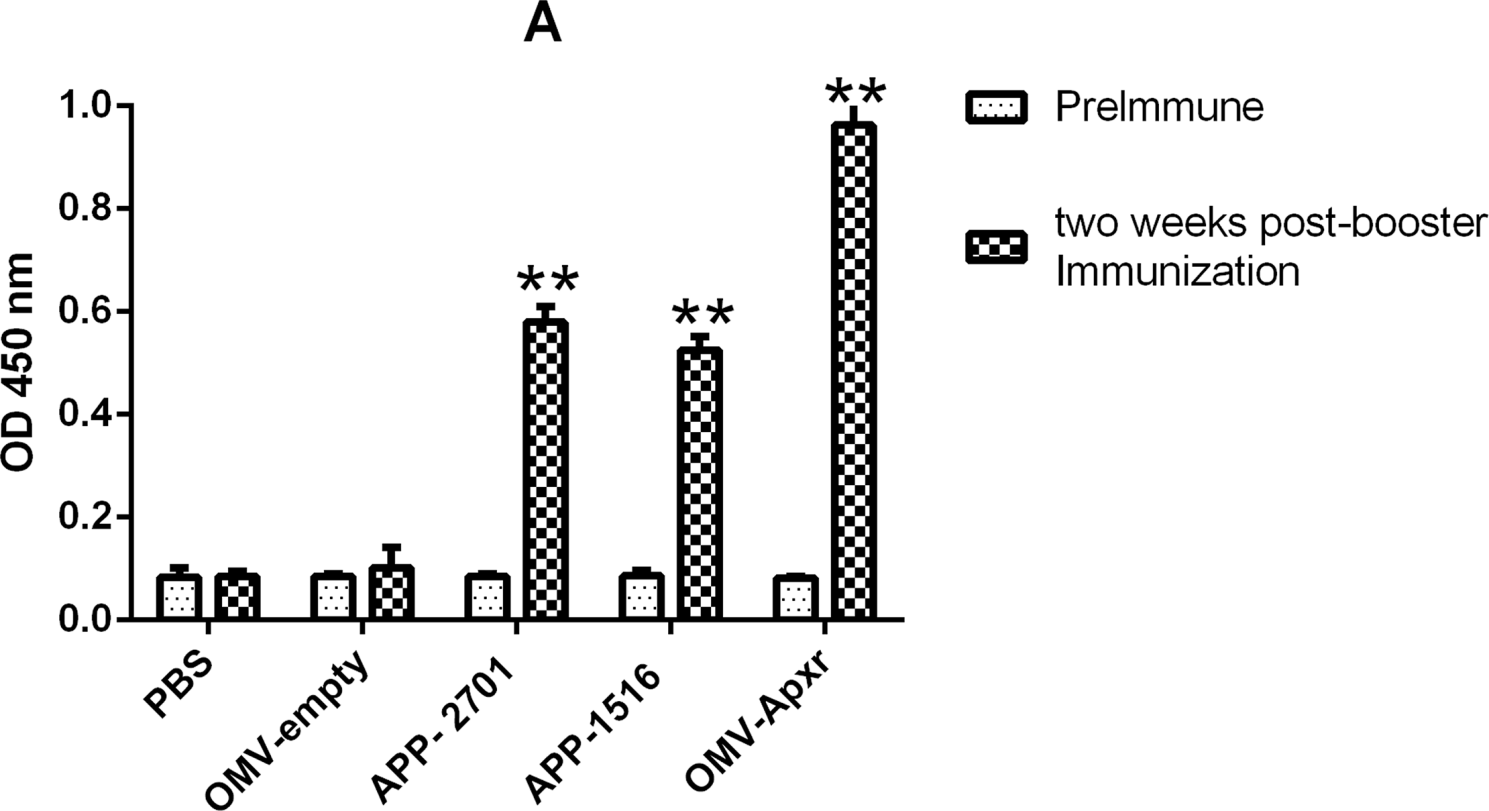
Analysis of levels of IgG. Serum samples from immunized controls and negative controls were collected before immunization and two weeks after booster immunization.

Apxr-OMVs vaccinated mice developed high anti-Apxr IgG titers 6 weeks post initial vaccination that were significantly greater than those of the sham negative control group (Fig 9, *P* < 0.05). IgG levels of the vaccinated group increased sharply post vaccination compared with the serum prior to immunization. The results showed that the Apxr-OMVs can generate a robust humoral immune response.

**Fig 10:**
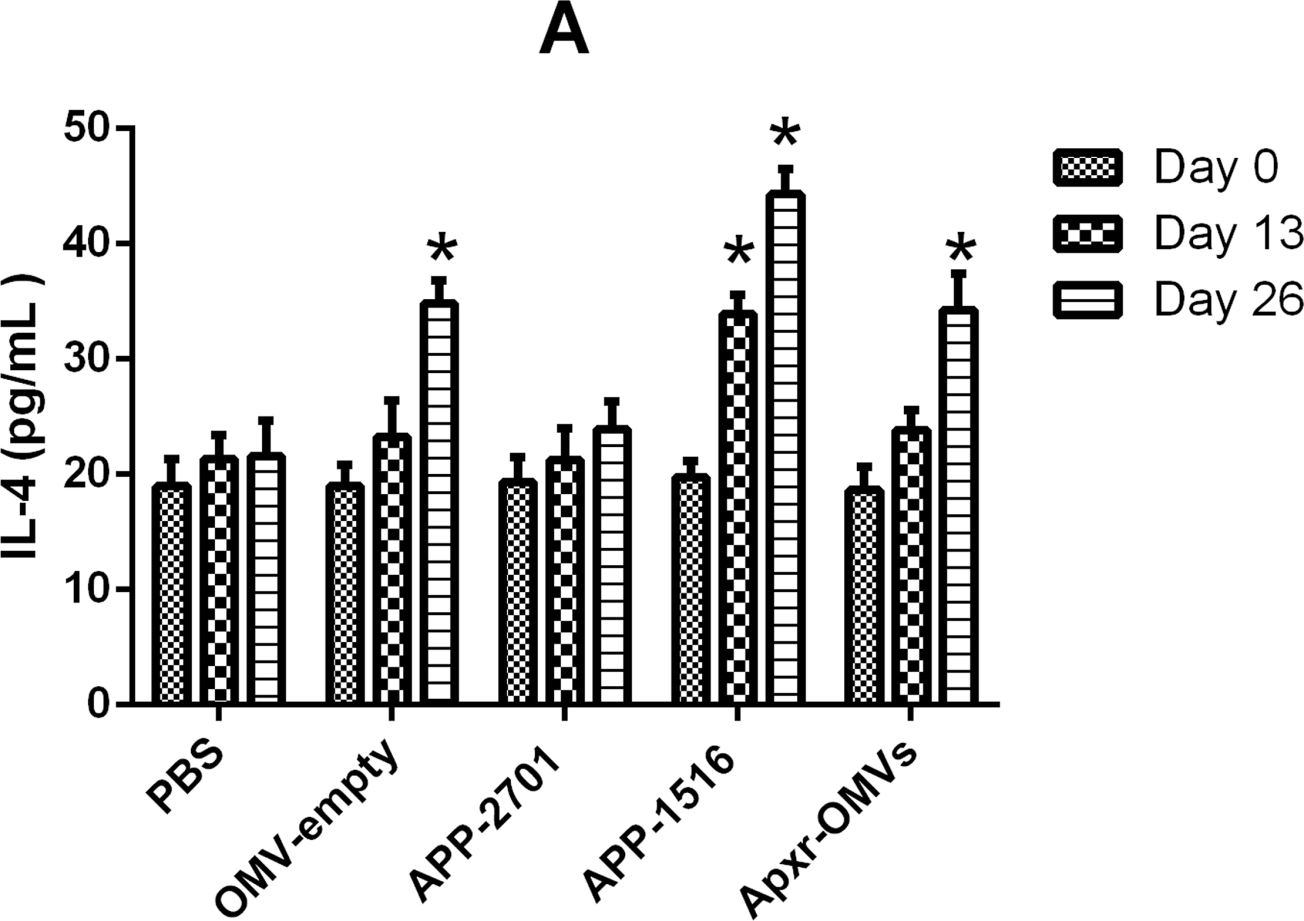
Cytokines detection assay. The levels of IL-4 against OMV-empty, APP-2701, APP-1516 and Apxr-OMVs in sera.

**Fig 11:**
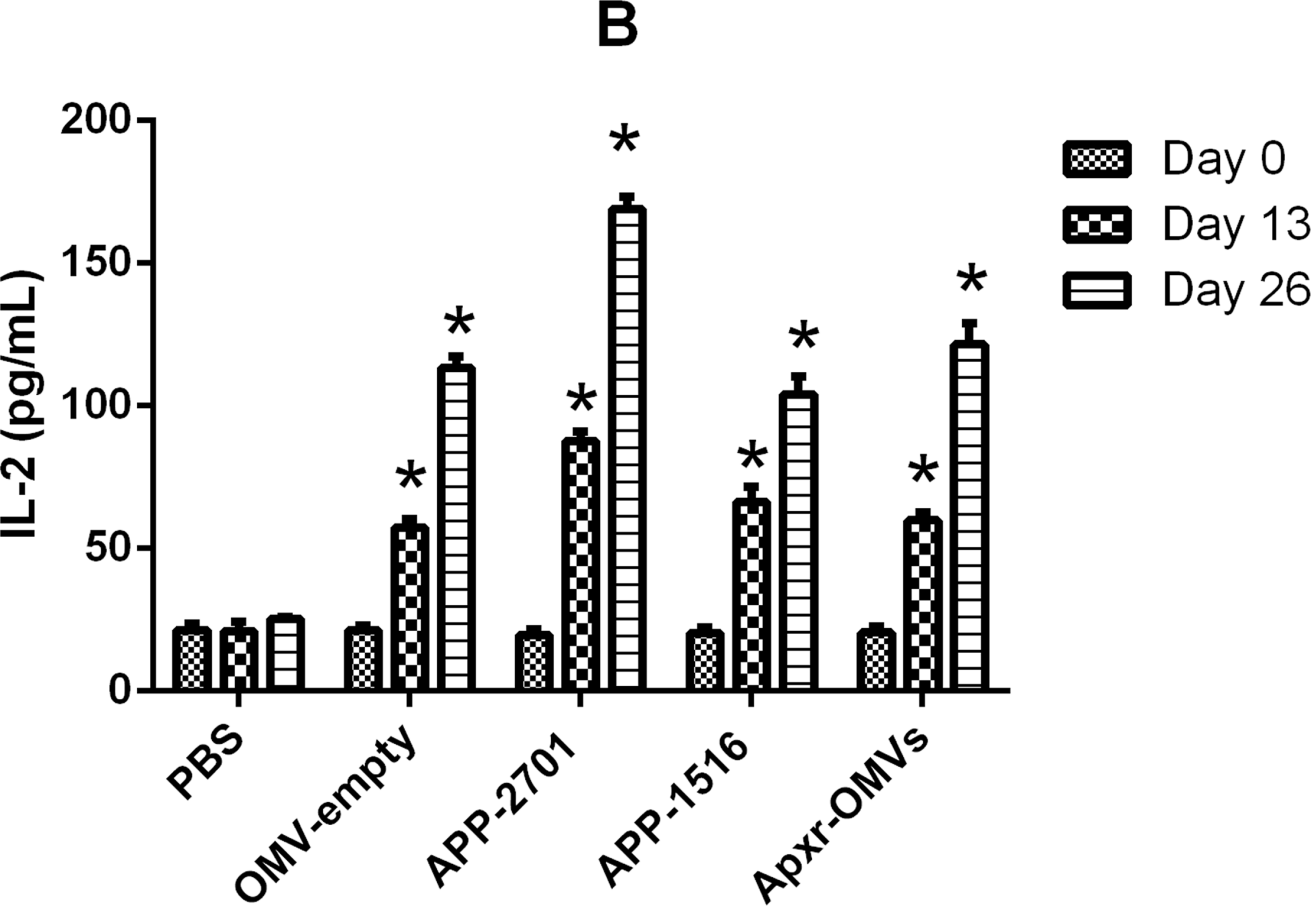
Cytokines detection assay. The levels of IL-2 against OMV-empty, APP-2701, APP-1516 and Apxr-OMVs in sera.

**Fig 12:**
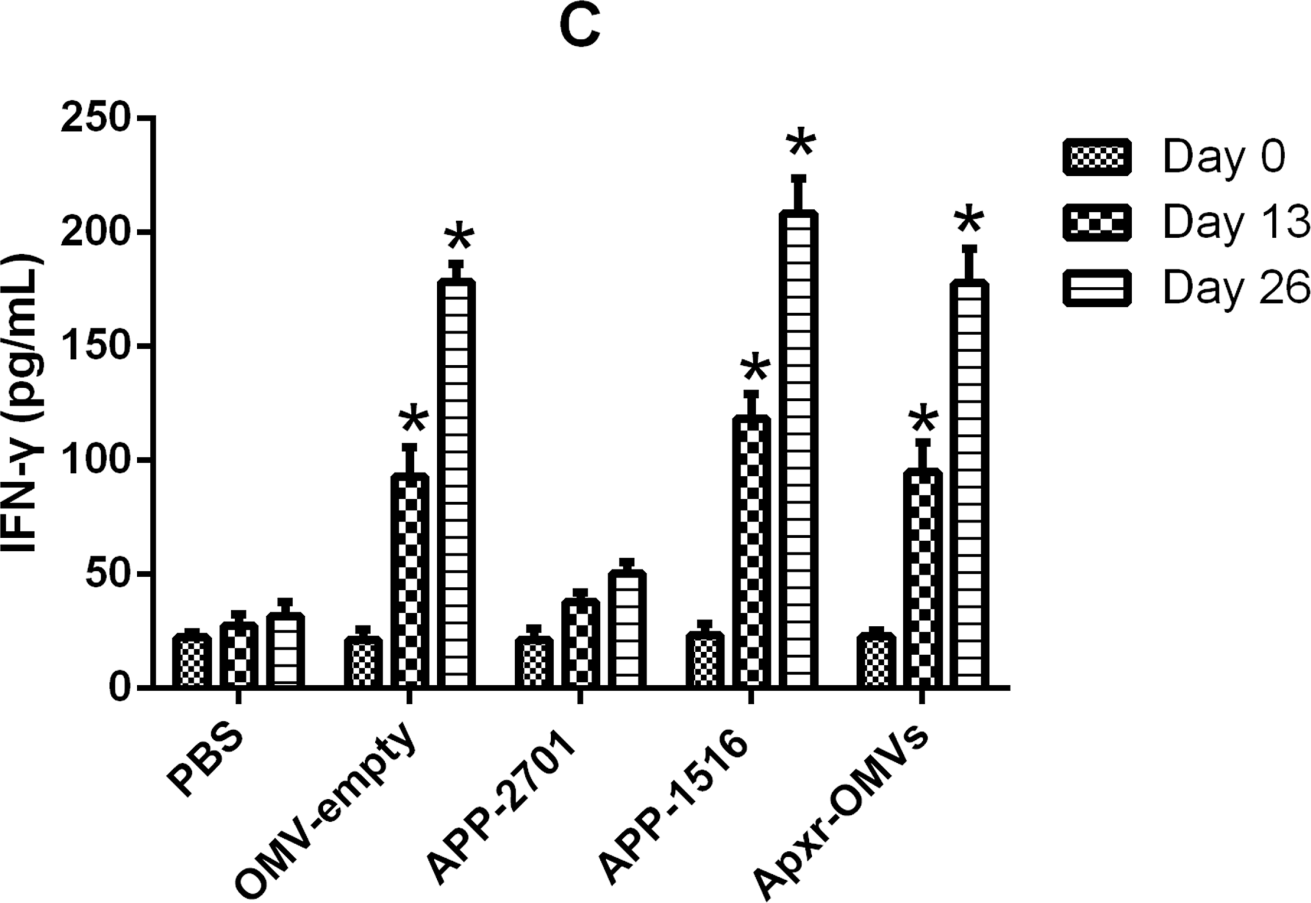
Cytokines detection assay. The levels of IFN-γ against OMV-empty, APP-2701, APP-1516 and Apxr-OMVs in sera.

On the other hand, the levels of IFN-γ, IL-2, IL-4 in the immunized groups were significant higher than the PBS and OMV-empty control groups (Figs 10–12, *P <* 0.05 =. Meanwhile, the results of the first and second immunization showed increasing levels of cytokines. The OMV-empty also promoted the generation of cytokines.

### Cell-mediated immune response

**Fig 13:**
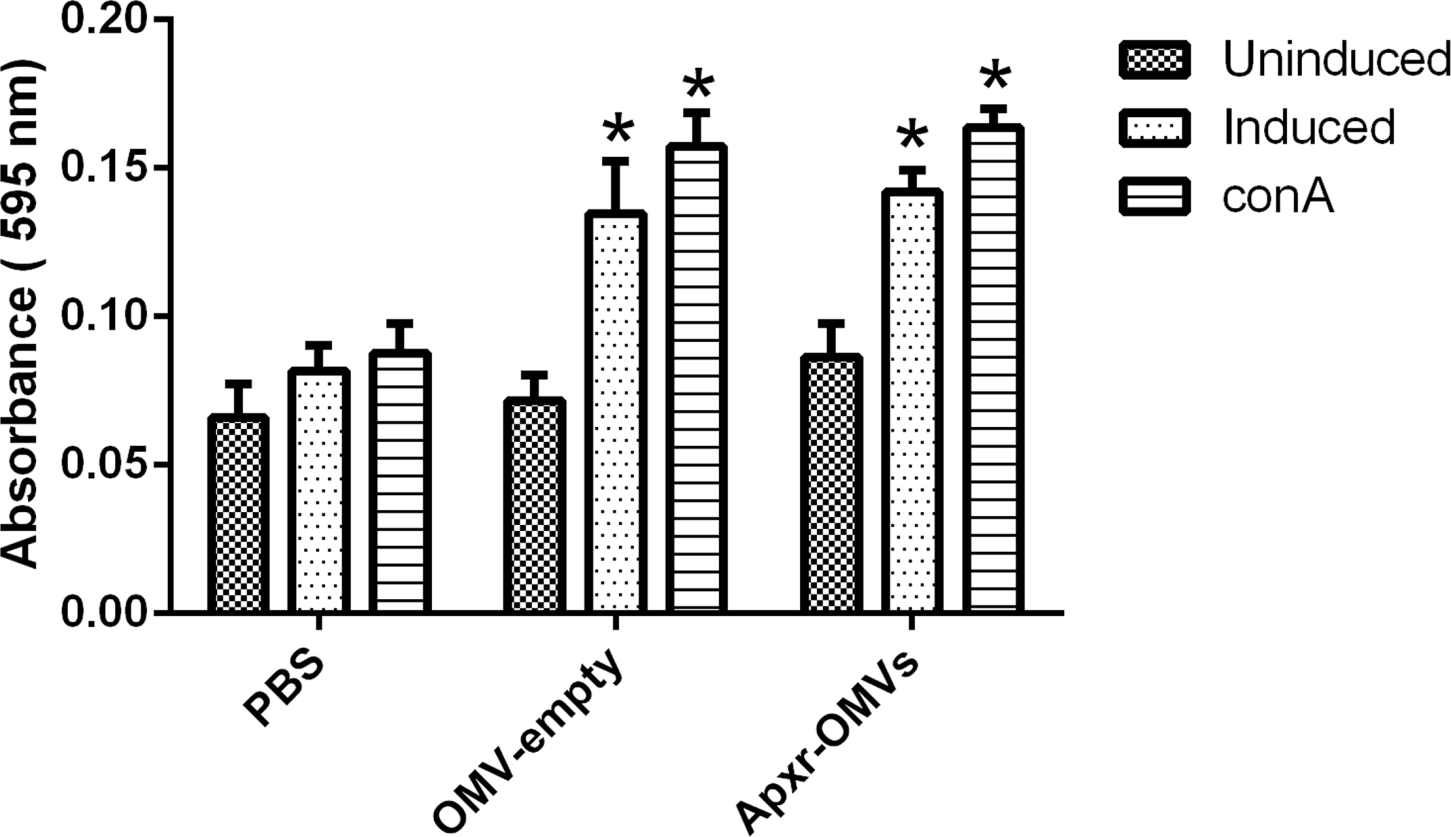
Lymphocyte proliferation assay. Levels of lymphocyte proliferation were detected using an MTS method and the results shown as absorbance at 490 nm. Splenocytes of mice two weeks post boost immunization were isolated and stimulated with recombinant Apxr protein and con A.

The levels of lymphocyte proliferation of the Apxr-OMVs group were higher than the PBS group (Fig 13, *P <* 0.05). Meanwhile, lymphocyte proliferation of Apxr-OMVs group was also detected after the cells were stimulated with ConA. The OMV-empty groups showed similar levels compared with Apxr-OMVs group (*P >* 0.05).

### OMV-Apxr protein protect BALB/c mice from APP challenge

**Fig 14:**
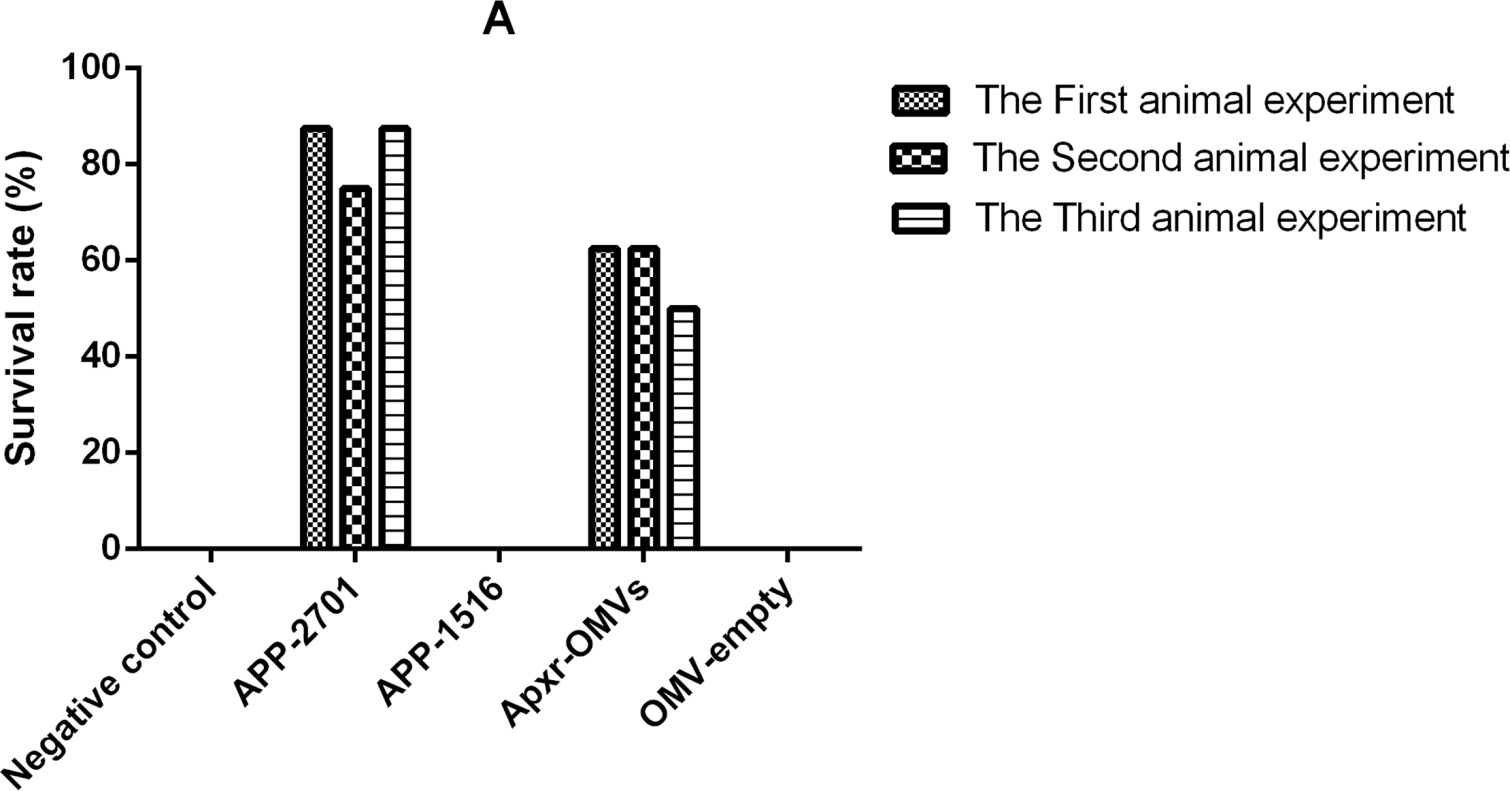
The results of repeated experiments. The Survival rates after APP strain 2701 of serotype 1 infection; n = 8 mice/ group. Animal experiments were repeated three times. Differences between trials were shown to be not significant (P > 0.05), indicating that the data is reliable.

**Fig 15:**
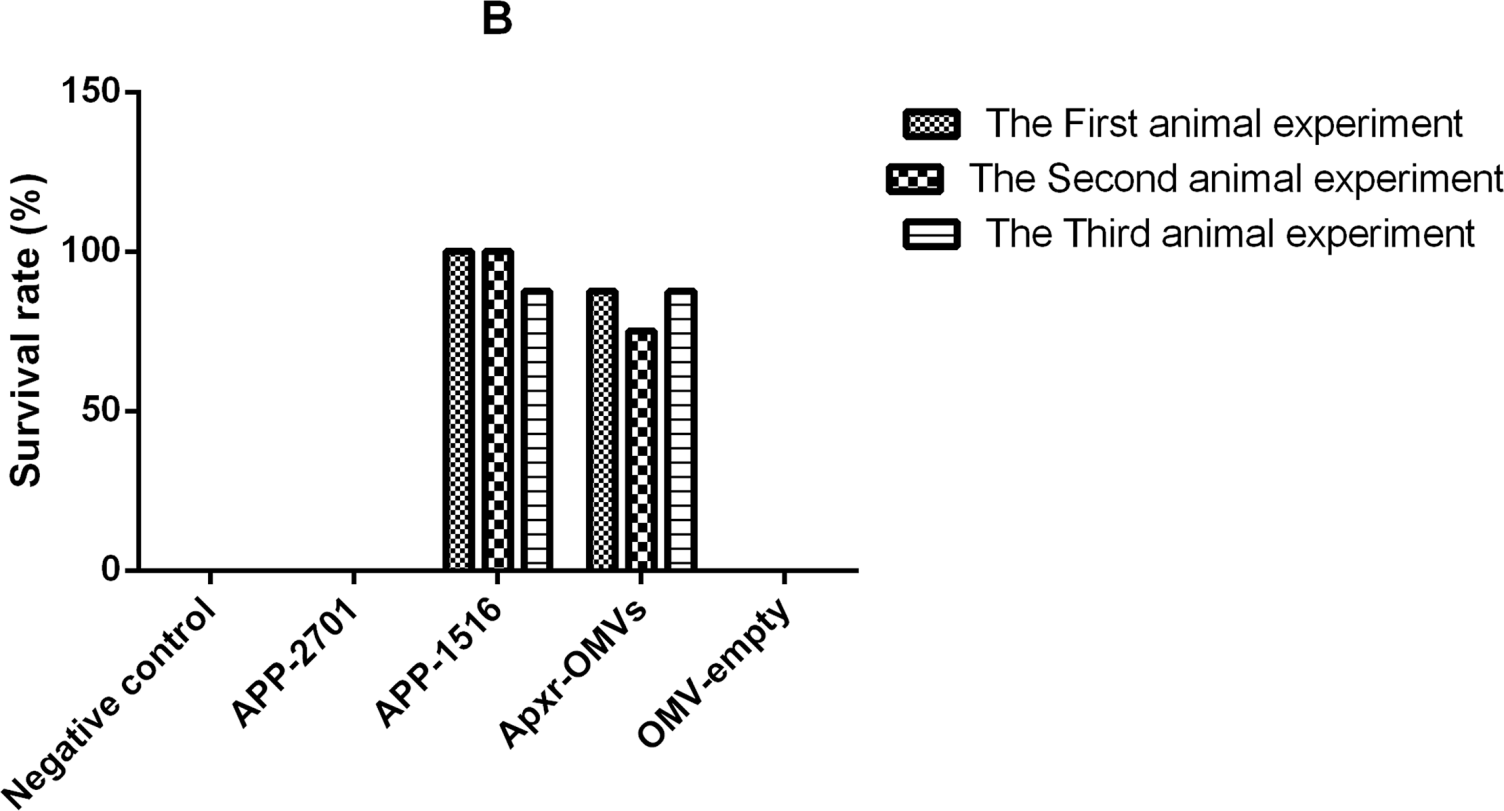
The results of repeated experiments. The Survival rates after APP strain 1516 of serotype 7 infection; n = 8 mice/ group. Animal experiments were repeated three times. Differences between trials were shown to be not significant (P > 0.05), indicating that the data is reliable.

All mice receiving PBS as control died within 48 hours after challenge with APP strain 2701and strain 1516 (Tables 2 and 3). The survival rates of Apxr-OMVs, APP-2701, APP-1516, OMV-empty and PBS groups were 62.5%, 87.5%, 0%, 0%, and 0%, respectively, when challenged with APP strain 2701. The survival rates of Apxr-OMVs, APP-1516, APP-2701, OMV-empty, and PBS groups were 87.5%, 100%, 0%, 0%, and 0%, respectively, when challenged with APP strain 1516. The organisms isolated from the dead mice were identified as APP with Colony morphology and PCR amplification [3]. There were no significant differences (P > 0.05) in the two replicates of animal experiments (Figs 14 and 15).

**Table 2:** Survival rates of mice challenged with APP strain 2701 of serotype 1 after immunization.

Group	1d	2d	3d	4d	5d	6d	7d
**Negtive**	**0%**	**0%**	**0%**	**0%**	**0%**	**0%**	**0%**
**APP-2701**	**87.5%**	**87.5%**	**87.5%**	**87.5%**	**87.5%**	**87.5%**	**87.5%**
**APP-1516**	**0%**	**0%**	**0%**	**0%**	**0%**	**0%**	**0%**
**Apxr-OMVs**	**62.5%**	**62.5%**	**62.5%**	**62.5%**	**62.5%**	**62.5%**	**62.5%**
**OMV-empty**	**0%**	**0%**	**0%**	**0%**	**0%**	**0%**	**0%**

**Table 3:** Survival rates of mice challenged with APP strain 1516 of serotype 7 after immunization.

Group	1d	2d	3d	4d	5d	6d	7d
**Negtive**	**0%**	**0%**	**0%**	**0%**	**0%**	**0%**	**0%**
**APP-2701**	**12.5%**	**0%**	**0%**	**0%**	**0%**	**0%**	**0%**
**APP-1516**	**100%**	**100%**	**100%**	**100%**	**100%**	**100%**	**100%**
**Apxr-OMVs**	**100%**	**87.5%**	**87.5%**	**87.5%**	**87.5%**	**87.5%**	**87.5%**
**OMV-empty**	**0%**	**0%**	**0%**	**0%**	**0%**	**0%**	**0%**

### Histopathologic analysis

**Fig 16:**
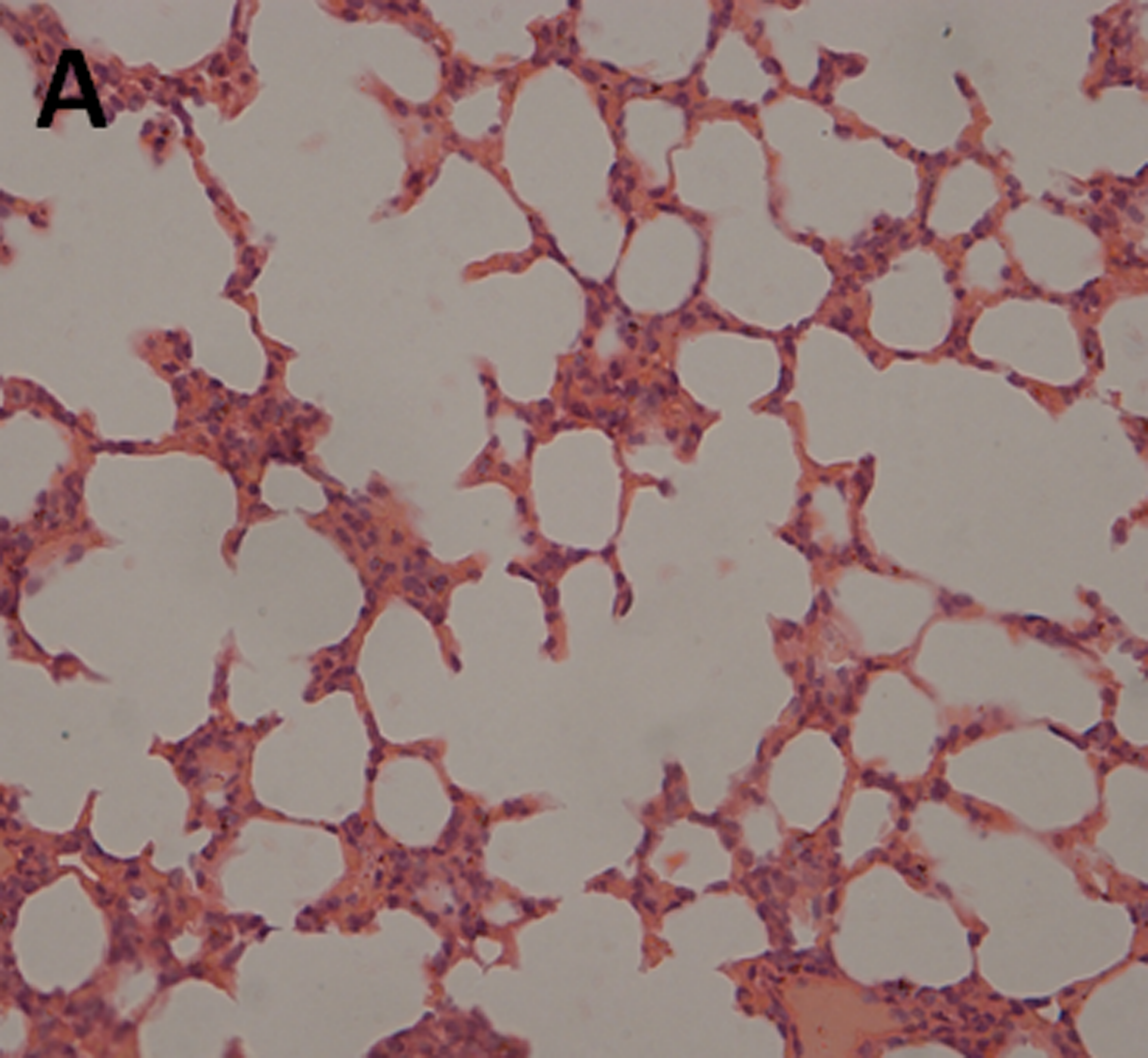
Lung Histopathology from normal control. H&E, Magnification 400 X.

**Fig 17:**
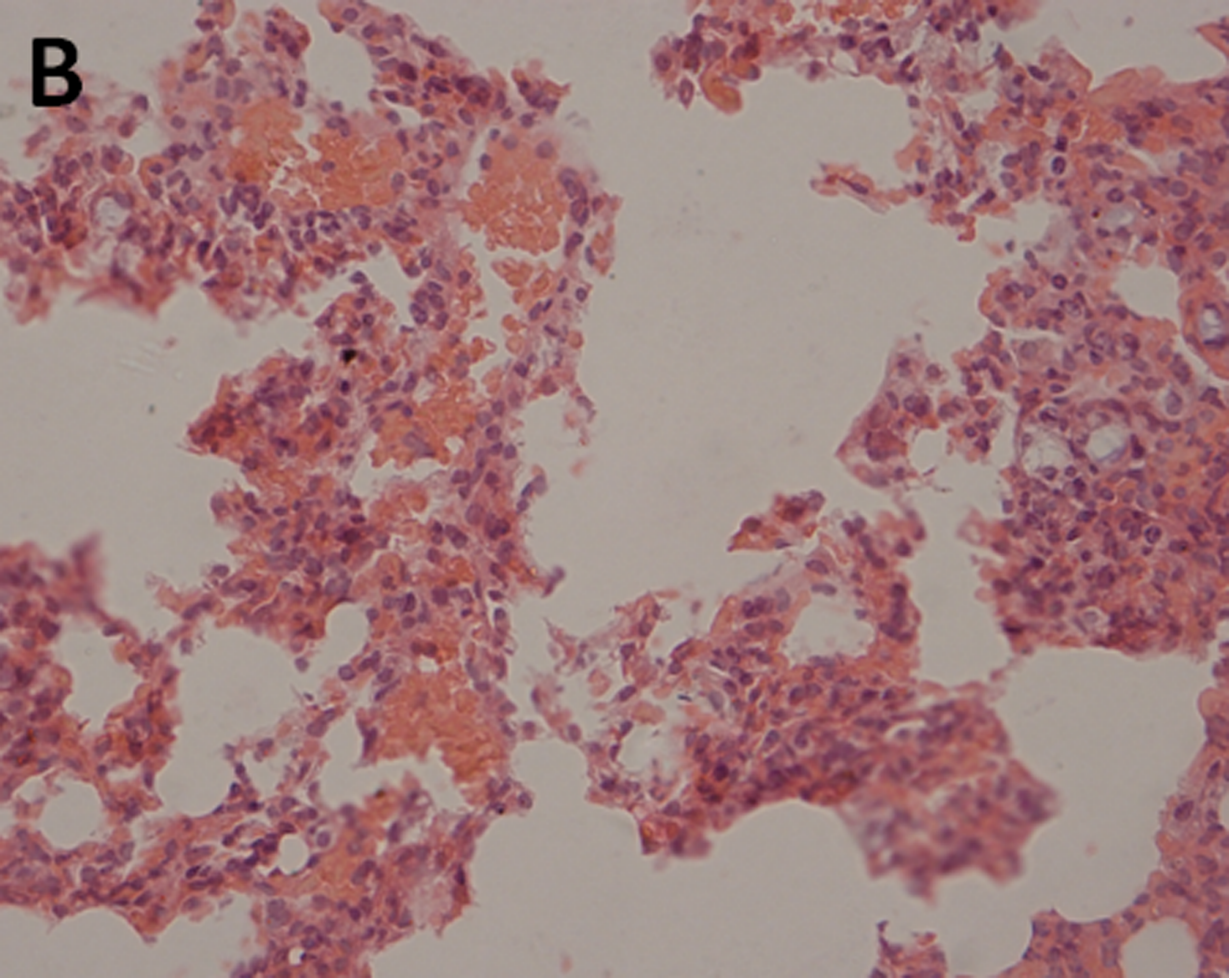
Lung Histopathology from mice immunized with PBS challenged by APP strain 2701. H&E, Magnification 400 X. Negative control mouse lung showing increased miscibility and inflammatory cell infiltration in the perivascular and peribronchial areas.

**Fig 18:**
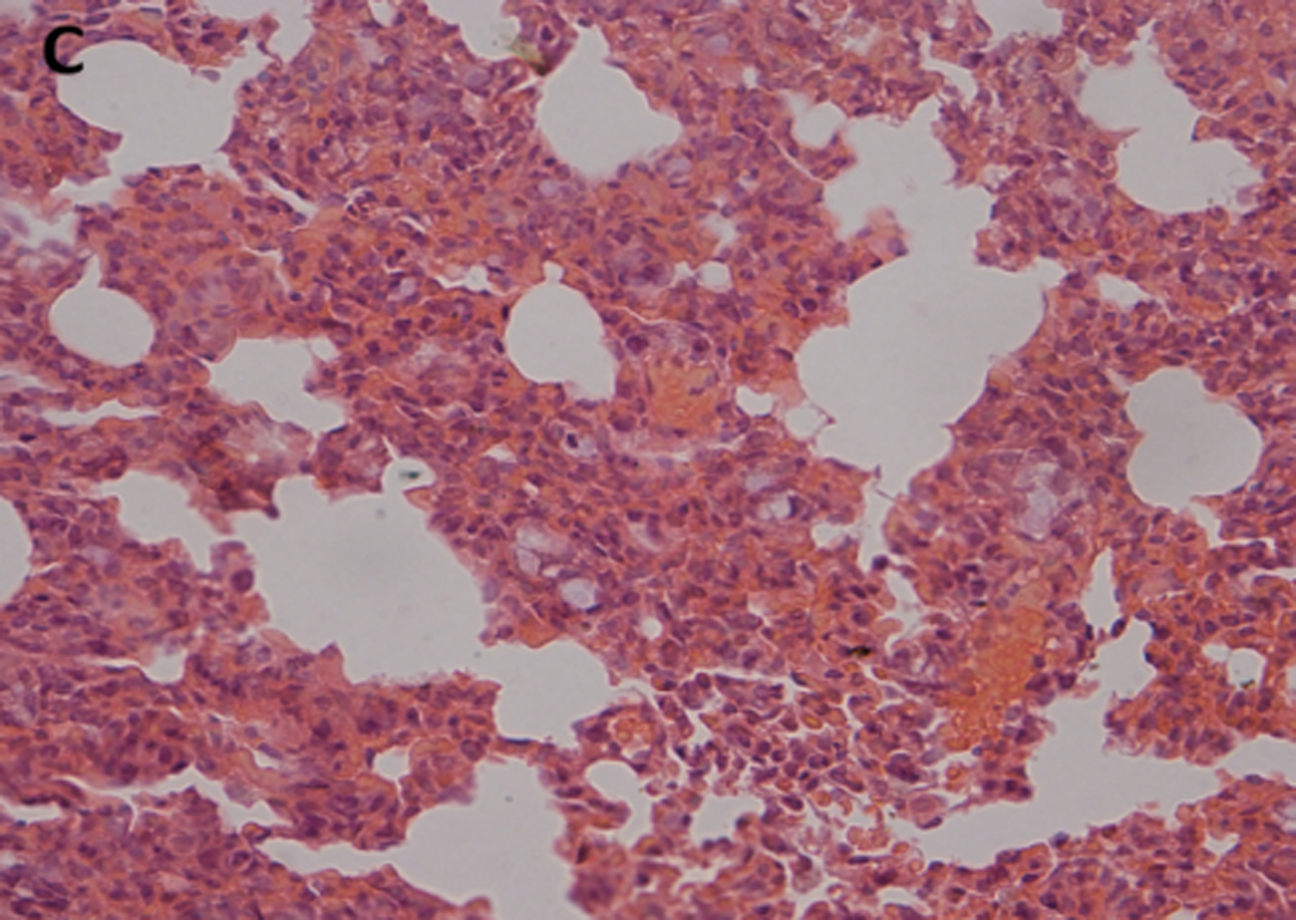
Lung Histopathology from mice immunized with PBS challenged by APP strain 1516. H&E, Magnification 400 X. Negative control mouse lung showing increased miscibility and inflammatory cell infiltration in the perivascular and peribronchial areas.

**Fig 19:**
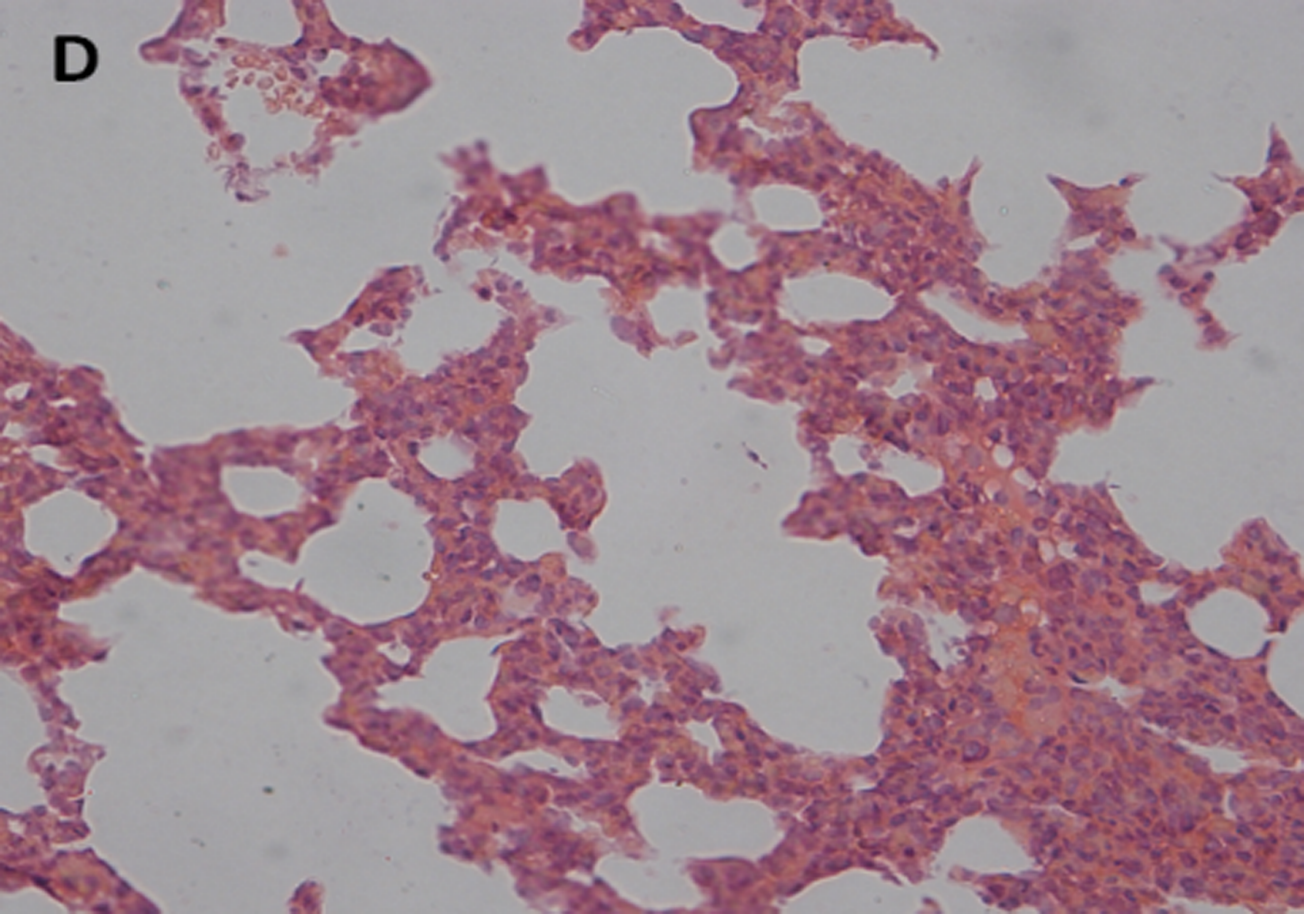
Lung Histopathology from mice immunized with OMV-Apxr challenged by APP strain 2701. H&E, Magnification 400 X. Lung tissue from mice challenged by APP strain 2701 showed a mild inflammatory cell infiltration in the perivascular and peribronchial areas.

**Fig 20:**
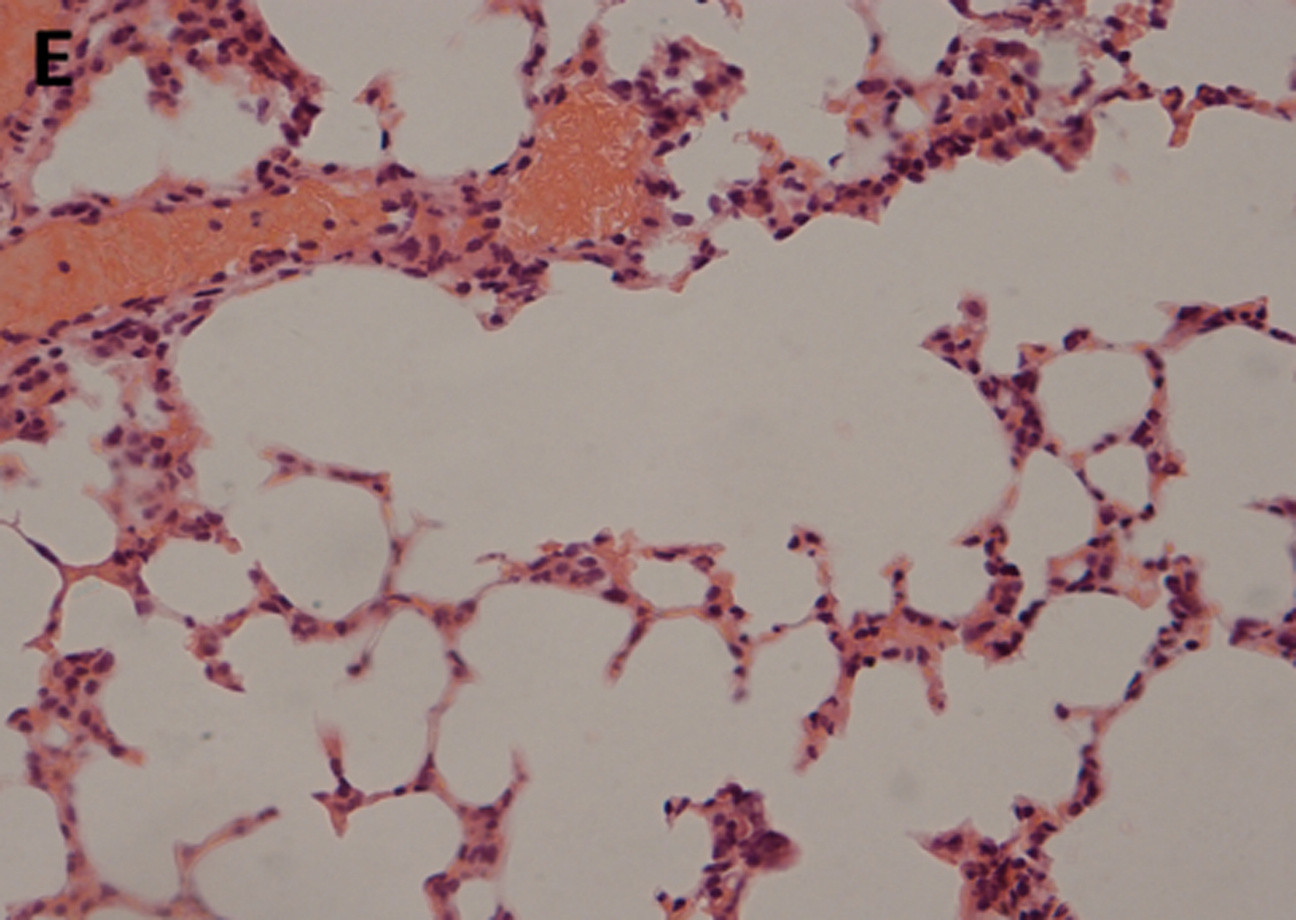
Lung Histopathology from mice immunized with OMV-Apxr challenged by APP strain 1516. H&E, Magnification 400 X. Lung tissue from surviving infected mice showing significantly reduced infiltration of inflammatory cells.

The histopathological examination of lung tissues showed infiltration of neutrophils and macrophages. The lung tissue from the control groups (PBS) of mice showed severe clinical symptoms after challenged with both serotypes of APP (Figs 16 and 17). The lungs displayed severe pulmonary interstitial edema, hemorrhage, hyperemia and inflammatory cells infiltration associated with alveolar atrophy and alveolar septum enlargement. The lung tissue damage levels in Apxr-OMVs vaccinated groups challenged with APP strain 2701 (Fig 18) or strain 1516 (Fig 19) were lower than in the control group (Fig 20). It indicates that the pathological changes were less evident in Apxr-OMVs vaccinated group challenged with APP strain 1516 or strain 2701 than in the PBS control group. We therefore concluded that Apxr-OMVs induced protection in mice against APP strain 1516 and strain 2701.

## Discussion

APP is the etiological agent of porcine pleuropneumonia (PCP), a significant disease that causes serious economic losses to the swine industry worldwide [29]. Vaccines appear to be the most effective choice for controlling PCP. However, the traditional inactivated whole-cell vaccines lack cross-protection against different serotypes of APP, and cannot reduce the infection rate of swine and resistance to pathogen colonization in the lungs [30]. Subunit vaccines greatly improve cross protection against different serotypes, however, the production process is cumbersome and with high costs hindering the commercialization process [31]. The development of safe, efficient and inexpensive new vaccines will become a more effective means of disease prevention and control. Compared to traditional Vaccine modes, OMV delivery systems have many potential advantages, including fewer applications, better safety, higher immune efficiency, adjuvant functions provided by the OMV nanoscale structure, LPS, and immune-stimulating molecules [32]. In addition, OMVs are resistant to expression of exogenous proteins and carry immune stimulators [33]. Increasingly, the innovation of synthetic or biologically derived nanoparticle antigen carriers has been successful [34], allowing for more efficient and targeted dissemination of antigens to key immune cell populations [35].

The current APP subunit vaccines derive primarily from Apx, a key virulence factors of APP. The glycine-Aspartic-rich nonapeptide repeats area in the C-terminal part of the ApxA protein secondary structure is predominantly β-folded and irregularly curled, possibly hydrophilic, and is highly immunogenic [9].The bioinformatics analysis of the Apxr fusion protein further confirms its immunogenic qualities.

In this study, we treated Apxr-OMVs with PK and EDTA and found that Apxr fusion proteins were displayed on the surface of recombinant OMVs. Our findings were consistent with those of a previous study [36,37]. We demonstrated that immunization with 80 μg Apxr-OMVs rapidly produced significantly higher Apxr-specific antibody responses than immunization with 80 μg OMVs-empty, a finding consistent with a previous report [26]. This study’s results, along with others indicate that the recombinant proteins comprises approximately 0.32% to 1% of the total protein content in the engineered OMVs [38,39]. This clearly showed OMVs to be vaccine carriers that are highly efficient at inducing low-dose heterologous protein humoral immune responses. OMVs have shown great potential as convenient and efficient antigen delivery platforms for novel multivalent vaccines.

The prevalent serotypes of APP in China are 1 and 7, APP strain 2701 of serotype 1 and APP strain 1516 of serotype 7 were chosen for our challenge study, and the Apxr-OMVs generated in our study elicit high levels of immune cross-protection in mice and could be considered as potential vaccine candidates for further study in piglets.

Levels of IL-2, IL-4, and IFN-γ in animals vaccinated with Apxr-OMVs protein or OMVs-empty protein were elevated, and results showed that Apxr-OMVs or OMVs-empty we applied could induce strong cellular immune responses within treated animals. Immunization of inactivated strain (positive control) produce higher level of IL4 and IL2, which may due to complex components of bacterin. It stimulates both humoral immune response and cellular immune response in the host.

Cytokines are soluble signal proteins produced by a variety of host cells (B lymphocyte, T lymphocyte, neutrophils, monocytes and macrophages) that have been stimulated by foreign antigens to regulate immune responses [40]. Helper T cells (Th cells) (divided into Th1 and Th2) interact with key cytokines, as these cells lines possess corresponding cytokine receptors. Cytokines IL-2 and IFN-γ trigger Th1 activity, and IL-4 stimulates Th2 responses [41]. IFN-γ is secreted by NK cells and T cells, and plays a central role in the immune response [42]. IL-4 can improve the level of antibody and strengthen inflammatory responses [43]. IL-2 can activate natural killer (NK) cells and lymphatic factor activated killer (LAK) cells and stimulate monocyte growth [36]. Our results showed that increased level of cytokines (IFN-γ) were detected in Apxr-OMVs or OMVs-empty vaccinated group, indicating a Th1 cell response. The results were consistent with recent studies [37, 38]. The lipid bilayer-nanometer structure presumably helps OMVs to freely shuttle into immune cells, thereby stimulating strong immune responses.

## Conclusion

In summary, OMVs can display fusion Apxr proteins on OMVs surfaces; immunization with Apxr-OMVs produced a strong Apxr-specific humoral immunity response in mice, without the use of aluminum adjuvants; and Apxr-OMVs immunized mice have been protected against lethal challenge with clinically isolated APP strains 2701 of serotype 1 and strain 1516 of serotype 7. In contrast, the OMV-empty and PBS (as negative controls) or inactivated strain of APP-2701 and APP-1516 (as positive controls) in the animal study were shown to not provide protection or cross-protection against APP challenge. The results indicated that Apxr-OMVs can be used a cross-protective vaccine against APP serotype 1 and 7 in mice and further studies focused on evaluating the ability of Apxr-OMVs to protect piglets against APP infection are in progress in our laboratory.

## Supporting information

S1 Table The sequences of *ClyA-ApxIAr-ApxIIAr-ApxIIIAr-His* fusion genes. The *E*. *coli ClyA* sequences is marked as Yellow; *A*. *pleuropneumoniae ApxIAr* sequences is marked green; *A*. *pleuropneumoniae ApxIIAr* sequences is marked as blue; *A*. *pleuropneumoniae ApxIIIAr* sequences is marked as red; His tag is marked as gray, and the final is stop codon.
(DOCX)
