# Supplementary material for: A trivalent Apx-fusion protein delivered by E. coli outer membrane vesicles induce protection against Actinobacillus pleuropneumoniae of serotype 1 and 7 challenge in a murine model
Source: PLoS One. 2018 Jan 26;13(1):e0191286. doi: 10.1371/journal.pone.0191286 (PMC5786296; doi:10.1371/journal.pone.0191286)
Supplement: S1 Table — The E. coli ClyA sequences is marked as Yellow; A. pleuropneumoniae ApxIAr sequences is marked green; A. pleuropneumoniae ApxIIAr sequences is marked as blue; A. pleuropneumoniae ApxIIIAr sequences is marked as red; His tag is marked as gray, and the final is stop codon. (DOCX) [file pone.0191286.s001.docx]

Supplement Table. . The sequences of *ClyA-ApxIAr-ApxIIAr-ApxIIIAr-His* fusion genes. The *E. coli* *ClyA* sequences is marked as Yellow; *A. pleuropneumoniae* *ApxIAr* sequences is marked green; *A. pleuropneumoniae* *ApxIIAr* sequences is marked as blue; *A. pleuropneumoniae* *ApxIIIAr* sequences is marked as red; His tag is marked as gray, and the final is stop codon.

ATGACTGAAATCGTTGCAGATAAAACGGTAGAAGTAGTTAAAAACGCAATCGAAACCGCAGATGGAGCATTAGATCTTTATAATAAATATCTCGATCAGGTCATCCCCTGGCAGACCTTTGATGAAACCATAAAAGAGTTAAGTCGCTTTAAACAGGAGTATTCACAGGCAGCCTCCGTTTTAGTCGGCGATATTAAAACCTTACTTATGGATAGCCAGGATAAGTATTTTGAAGCAACCCAAACAGTGTATGAATGGTGTGGTGTTGCGACGCAATTGCTCGCAGCGTATATTTTGCTATTTGATGAGTACAATGAGAAGAAAGCATCCGCCCAGAAAGACATTCTCATTAAGGTACTGGATGACGGCATCACGAAGCTGAATGAAGCGCAAAAATCCCTGCTGGTAAGCTCACAAAGTTTCAACAACGCTTCCGGGAAACTGCTGGCGTTAGATAGCCAGTTAACCAATGATTTTTCAGAAAAAAGCAGCTATTTCCAGTCACAGGTAGATAAAATCAGGAAGGAAGCATATGCCGGTGCCGCAGCCGGTGTCGTCGCCGGTCCATTTGGATTAATCATTTCCTATTCTATTGCTGCGGGCGTAGTTGAAGGAAAACTGATTCCAGAATTGAAGAACAAGTTAAAATCTGTGCAGAATTTCTTTACCACCCTGTCTAACACGGTTAAACAAGCGAATAAAGATATCGATGCCGCCAAATTGAAATTAACCACCGAAATAGCCGCCATCGGTGAGATAAAAACGGAAACTGAAACAACCAGATTCTACGTTGATTATGATGATTTAATGCTTTCTTTGCTAAAAGAAGCGGCCAAAAAAATGATTAACACCTGTAATGAGTATCAGAAAAGACACGGTAAAAAGACACTCTTTGAGGTACCTGAAGTCTATGCGGGTAACGGACATGATGTAGCATATTACGATAAAACCGATACAGGTTACTTAACATTTGACGGACAAAGTGCACAGAAAGCCGGTGAATATATTGTCACTAAAGAACTTAAAGCTGATGTAAAAGTTTTAAAAGAAGTGGTTAAAACTCAGGATATTTCAGTTGGAAAACGCAGTGAAAAATTAGAATATCGTGATTATGAGTTAAGCCCATTCGAACTTGGGAACGGTATCAGAGCTAAAGATGAATTACATTCTGTTGAAGAAATTATCGGTAGTAATCGTAAAGACAAATTCTTTGGTAGTCGCTTTACCGATATTTTCCATGGTGCGAAAGGCGATGATGAAATCTACGGTAATGACGGCCACGATATCTTATACGGAGACGACGGTAATGATGTAATCCATGGCGGTGACGGTAACGACCATCTTGTTGGTGGTAACGGAAACGACCGATTAATCGGCGGAAAAGGTAATAATTTCCTTAATGGCGGTGATGGTGACGATGAGTTGCAGGTCTTTGAGGGTCAATACAACGTATTATTAGGTGGTGCGGGTAATGACATTCTGTATGGCAGCGATGGTACTAACTTATTTGACGGTGGTGTAGGCAATGACAAAATCTACGGTGGTTTAGGTAAGGATATTAATTTAGGTGCTGGTAACGATAATGTATTTGTTGGGTCAAGTACTACCGTTATTGATGGCGGGGACGGACATGATCGAGTTCACTACAGTAGAGGAGAATATGGCGCATTAGTTATTGATGCTACAGCCGAGACAGAAAAAGGCTCATATTCAGTAAAACGCTATGTCGGAGACAGTAAAGCATTACATGAAACAATTGCCACCCACCAAACAAATGTTGGTAATCGTGAAGAAAAAATTGAATATCGTCGTGAAGATGATCGTTTTCATACTGGTTATACTGTGACGGACTCACTCAAATCAGTTGAGGAGATCATTGGTTCACAATTTAATGATATTTTCAAAGGAAGCCAATTTGATGATGTGTTCCATGGTGGTAATGGTGTAGACACTATTGATGGTAACGATGGTGACGATCATTTATTTGGTGGCGCAGGCGATGATGTTATCGATGGAGGAAACGGTAACAATTTCCTTGTTGGAGGAACCGGTAATGATATTATCTCGGGAGGTAAAGATAATGATATTTATGTCCATAAAACAGGCGATGGAAATGATTCTATTACAGACTCTGGCGGACAAGATAAACTGGCACATCTAGGCAATGGTAATGACAAAGTGTTCTTAGCTGCGGGTTCCGCAGAAATTCACGCTGGTGAAGGTCATGATGTGGTTTATTATGATAAAACCGATACAGGTCTTTTAGTAATTGATGGAACCAAAGCGACTGAACAAGGGCGTTATTCTGTTACGCGCGAATTGAGTGGTGCTACAAAAATCCTGAGAGAAGTAATAAAAAATCAAAAATCTGCTGTTGGTAAACGTGAAGAAACCTTGGAATATCGTGATTATGAATTAACGCAATCAGGTAATAGTAACCTAAAAGCACATGATGAATTACATTCAGTAGAAGAAATTATTGGAAGTAATCAGAGAGACGAATTTAAAGGTAGTAAATTCAGAGATATTTTCCATGGTGCCGATGGTGATGATCTATTAAATGGTAATGATGGGGATGATATTCTATACGGTGATAAAGGTAACGATGAGTTAAGAGGTGATAACGGTAACGACCAACTTTATGGTGGTGAAGGTGATGACAAACTATTAGGAGGTAATGGCAATAATTACCTCAGTGGTGGTGATGGCAATGATGAGCTTCAAGTATTAGGCAATGGTTTTAATGTGCTTCGTGGCGGTAAAGGCGATGATAAACTTTATGGTAGCTCAGGTTCTGATTTACTTGATGGTGGAGAAGGTAATGATTATCTAGAAGGAGGCGATGGTAGCGATTTTCACCACCACCACCACCACTAA
